# The Oncogenic Relevance of miR-17-92 Cluster and Its Paralogous miR-106b-25 and miR-106a-363 Clusters in Brain Tumors

**DOI:** 10.3390/ijms19030879

**Published:** 2018-03-16

**Authors:** Renata Gruszka, Magdalena Zakrzewska

**Affiliations:** Department of Molecular Pathology and Neuropathology, Medical University of Lodz, Pomorska 251, 92-213 Lodz, Poland; magdalena.zakrzewska@umed.lodz.pl

**Keywords:** brain tumor, cluster, microRNA, miR-17-92, miR-106b-25, miR-106a-363, nervous system, OncomiR-1

## Abstract

The fundamental function of ribonucleic acids is to transfer genetic information from DNA to protein during translation process, however, this is not the only way connecting active RNA sequences with essential biological processes. Up until now, many RNA subclasses of different size, structure, and biological function were identified. Among them, there are non-coding single-stranded microRNAs (miRNAs). This subclass comprises RNAs of 19–25 nucleotides in length that modulate the activity of well-defined coding RNAs and play a crucial role in many physiological and pathological processes. miRNA genes are located both in exons, introns, and also within non-translated regions. Several miRNAs that are transcribed from the adjacent miRNA genes are called cluster. One of the largest ones is miR-17-92 cluster known as OncomiR-1 due to its strong link to oncogenesis. Six miRNAs from the OncomiR-1 have been shown to play important roles in various physiological cellular processes but also through inhibition of cell death in many cancer-relevant processes. Due to the origin and similarity of the sequence, miR-17-92 cluster and paralogs, miR-106b-25 and miR-106a-363 clusters were defined. Here we discuss the oncogenic function of those miRNA subgroups found in many types of cancers, including brain tumors.

## 1. Introduction

About 80% of the human genome is transcribed but only up to 2% of it comprises protein coding sequences. In spite of the progress of knowledge, the function of this huge part of genome is still undefined [1]. The main factors controlling the expression of genetic information during translation processes are ribosomal RNA (rRNA) and transfer RNA (tRNA). However, currently non-coding RNAs represent the most interesting regulators of gene activity. Within the group of non-coding RNAs there are some sequences which are expressed constitutively. They participate in basic processes related to gene expression and play an important role in proper functioning of the cell. In many biological processes non-coding RNAs are extremely versatile modulating genes expression at multiple levels—from chromatin structure modification, transcription, pre-mRNA sequencing, translation regulation, and transcriptional stability [2]. 

As a result, most of the transcript of human genome consists of sequences that have no protein coding capability. Non-coding RNA (ncRNA) includes small nuclear RNA (snRNA) and small nucleotide RNA (snoRNA), which regulate maturation and modification of primary transcripts, as well as a heterogeneous group of small regulatory RNAs (srRNAs) [3,4]. srRNAs are divided into three main groups: small interfering RNAs (siRNAs); microRNAs (miRNAs); and Piwi-interacting RNAs (piRNAs) [5,6]. 

Here we focused on miRNAs, a class of endogenous, non-coding single stranded RNAs of approximately 19–25 nucleotides in length, which modulates the activity of strictly defined RNA fragments and play a special role in many physiological and pathological processes [7]. Genes for miRNAs are often organized in clusters that are transcribed as polycistronic transcription units. They may exist between protein coding sequences where acting as standalone transcription units and also can be found in exons, introns, and untranslated regions [8]. Because of their extensive role in gene regulation, miRNAs are involved in many cellular processes, such as proliferation, development, differentiation, apoptosis, and tumor growth [7,9]. 

One of the most interesting is miR-17-92 cluster with its paralogs, miR-106b-25 and miR-106a-363 clusters described below. This review is focused on the role of those miRNA families during oncogenesis of the most frequent brain tumors and sympathetic nervous system tumors.

## 2. Biogenesis and Function of miRNA

**Figure 1:**
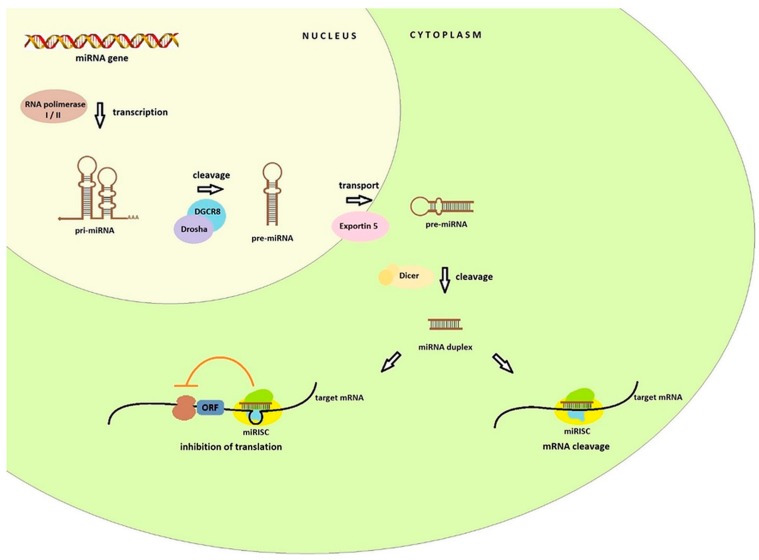
Pathway of miRNA biogenesis. In the nucleus RNA polymerase I or II synthesizes primary miRNA. Then, pri-miRNA are processed into pre-miRNA hairpins by the Drosha DGCR8 microprocessor. Pre-miRNA is exported into the cytoplasm by the transport protein Exportin 5. In the next step, the Dicer endonuclease create a miRNA duplex. The mature miRNA guides the RISC to target mRNA to inhibit the translation.

miRNA biogenesis starts in the nucleus (Figure 1) with the transcription of the miRNA genes by the RNA polymerase II (or RNA polymerase I in some human miRNAs). It results in the formation of hairpin loop primary transcript known as pri-miRNA, of over several kilobases long, which contains both a 5′ cap and a poly(A) tail. Nuclear protein DGCR8 recognizes double-stranded pri-miRNA structures. The DGCR8 is associated with the Drosha, RNA-specific nuclease. Together they form a microprocessor complex that take part in the processing of primary miRNA transcripts in the nucleus. DGCR8 in this complex attaches to the single stranded pri-miRNA ends and directs the catalytic ribonuclease domain so that it is cleaved releasing the miRNA precursor (pre-miRNA) hairpin structures of 60–100 nucleotides [10,11]. In this form, they are transferred to the cytoplasm by the transport proteins Exportin 5 (Exp-5). In the cytoplasm the Dicer endonuclease allows the formation of a miRNA duplex of approximately 22 nucleotides. One of the strands of the duplex (called the passenger strand or miR*) is discarded, second strand (called the guide strand, leading strand or miR) is connected to the RISC (RNA-induced silencing complex). The basic components of the RISC loading complex are Dicer, Ago2, PACT, and TRBP, among which the Ago2 protein has endonuclease activity. RISCs associated with miRNAs are designated miRNA containing ribonucleoprotein complex (miRISC or miRNP) [7,9,12]. This complex is directed by miRNA to the target mRNA. Crucial for target recognition is the seed region (sequence spanning from position 2 to 8 at the 5′ end) of the miRNA guide which target mRNA hybridizes through complementarity of the bases. The effect of this combination can be twofold: cleavage of target mRNA with subsequent degradation or translation inhibition and it is dependent on the degree of match between the miRNA and its target mRNA [12,13]. In the first case, the specific miRNA binds to cellular mRNAs containing sequences that are completely or almost completely complementary. The target mRNA is cleaved in the middle of the hybridizing sequence, while the miRNA itself remains unchanged and may re-participate in mRNA recognition and degradation. The level of the specified transcript and the synthesis of the protein that it encodes are lowered. This mechanism is commonplace in the world of plants [14,15].

In animals the regulation of gene expression does not depend on full complementarity between miRNA and target mRNA. Regulatory binding of miRNA usually occurs within 3′UTR, although miRNA binding sites have been shown to be present in the ORF (open reading frame) or 5′UTRs and the coding regions and leads to inhibition translation (at the translation initiation stage or post initiation stage), RNA destabilization, or a combination of these two activities [15,16,17,18]. miRNAs may be complementary to more than one mRNA fragment and conversely multiple miRNAs can cooperatively regulate a single mRNA target which exerts an influence on regulation of many cellular mechanisms [11,19].

## 3. Nomenclature of miRNA

According to the criteria for the annotation of the miRNA sequence, the names of the individual miRNAs are composed of the prefix “mir” and the identification number assigned chronologically [20]. Gene designations are created in a similar way, with the writing in italics. When the miRNA sequence concerned is part of a human genome, the prefix occurs in the form miR (miR-1); for sequences in genomes of other animals all letters are lowercase (mir-1), while capital letters are used for plants (MIR1). The primary miRNA transcripts are designated as pri-miR-1 (pri-mir-1, pri-MIR-1), the miRNA precursor as pre-miR-1 (pre-mir-1, pre-MIR-1) [21]. 

The rapid development of science in the field of miRNA resulted in the introduction of additional classification markings and systematization naming miRNAs. The miRNA naming was enriched with 3 or 4 letter prefixes to identify the species so that the identifiers were in the form of hsa-miR-1 for *Homo sapiens*, mmu-mir-1 for *Mus musculus*. Sequences of mature miRNAs differ at only one or two positions are given lettered suffixes, e.g., hsa-miR-1a and hsa-miR-1b. Distinct hairpin loci that give rise to identical mature miRNAs have numbered suffixes like hsa-miR-1-1 and hsa-miR-1-2 [22,23]. The result of pre-miRNA processing is to obtain two miRNAs of 19–25 nucleotides in length, one from each arm. Initially, the asterisk symbol was used to designate the less dominant miRNA form. However, the mature sequences from both arms of the hairpin can be biologically functional and therefore the miRNA names have been expanded by the suffixes-5p and -3p for sequences derived from the 5′ and 3′ arms of the hairpin precursor [24,25].

## 4. The miR-17-92 Cluster and Its Paralogs

**Figure 2:**
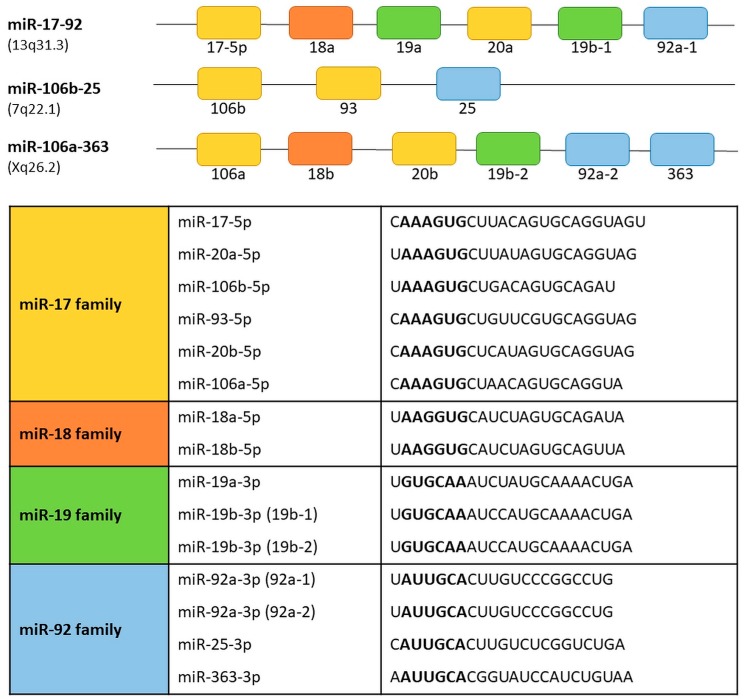
Transcript organization of the human miR-17-92 and its paralogs, miR-106a-363 and miR-106b-25 clusters. miRNAs sharing the same seed sequence are represented by boxes of the same color. The miRNAs are grouped into four seed families. Seed sequences are shown in bold.

The term “cluster” in relation to some miRNAs subgroups is used to underline their functional connection and/or genomic location. The name of the cluster applies to a group of two or more miRNAs that are transcribed from the physically adjacent miRNA genes, transcribed in the same orientation and not separated by the transcription unit or miRNA in the opposite orientation [26]. According to that the precursor transcript derived from the miR-17-92 gene includes six tandem stem-loop hairpin structures which results in six mature miRNAs: miR-17-5p, miR-18a, miR-19a, miR-20a, miR-19b-1, and miR-92a-1 [27,28,29,30]. The OncomiR-1 is grouped within an 800 base-pair region in the intron 3 in the locus of the non-protein-coding gene *MIR17HG/C13orf25* of human chromosome 13. The cluster is a prototypical example of a polycistronic miRNA gene. The miR-17-92 cluster has two paralogs: the miR-106b-25 and the miR-106a-363 (Figure 2). 

The miR-106b-25 cluster is located on chromosome 7 within the intron 13 region of the *MCM7* gene. The miR-106b-25 cluster encodes miR-106b, miR-93, and miR-25. The miR-106a-363 cluster is located on the X chromosome and contains six miRNAs: miR-106a; miR-18b; miR-19b-2; miR-20b; miR-92a-2; and miR-363 [31]. Reconstruction of the evolutionary history of the miR-17-92 complex and its paralogs demonstrated that they originate from tandem genetic duplication of individual members of clusters, followed by duplications of entire clusters and next loss of individual miRNAs [30,32]. Based on the sequence homology and seed conservation the miRNAs can be grouped into four miRNA families: the miR-17 family (miR-17-5p, miR-20a, miR-20b, miR-106a, miR-106b, miR-93); the miR-18 family (miR-18a, miR-18b); the miR-19 family (miR-19a, miR-19b-1 and miR-19b-2); and miR-92 family (miR-92a-1, miR-92a-2, miR-25, miR-363). The miR-17-92, miR-106b-25, and miR-106a-363 clusters act as oncogenes [30,33]. Their expression promotes cell proliferation, induces tumor angiogenesis, and suppresses apoptosis of cancer cells [33]. 

## 5. Regulation of miR-17-92 Cluster

**Figure 3:**
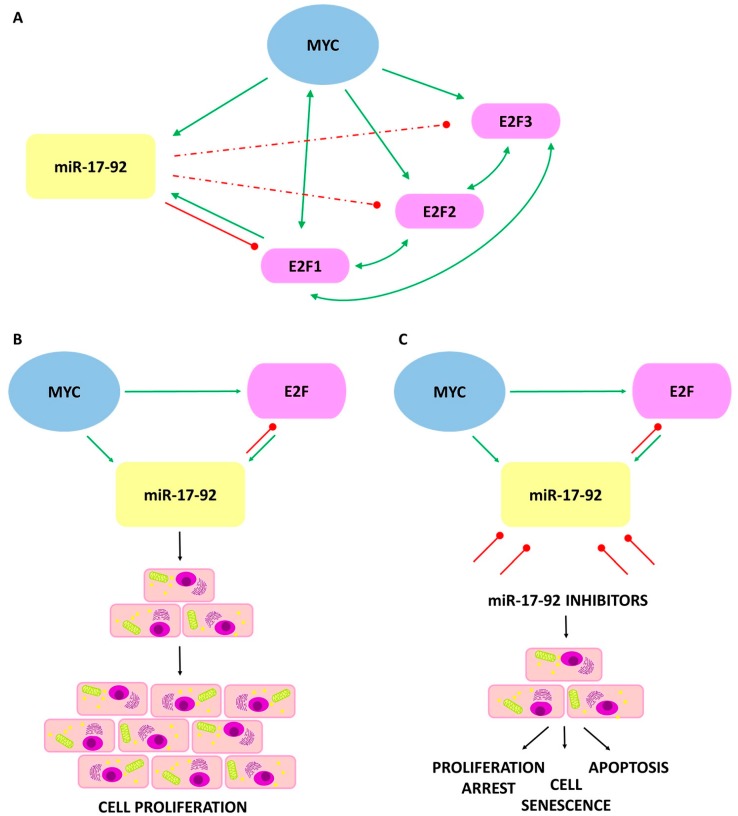
The interaction between MYC/E2F/miR-17-92 and its effect on the cancer cells. Green arrows indicate a transcriptional induction. Bidirectional arrows refer to mutual transcriptional induction. Red lines indicate translational inhibition, dashed lines indicate weaker inhibition. (**A**) The interactions among MYC, E2F1, E2F2, and E2F3 transcription factors and the miRNAs of the miR-17-92 cluster; (**B**) Elevated levels of miR-17-92 cluster cause disruption in homeostasis leading to a decrease in apoptotic ability and increasing the proliferation of tumor cells; (**C**) Inhibition of miR-17-92 activity stop excessive proliferation, restoring the process of cell aging and apoptosis.

In vitro studies have shown that MYC is a potent and direct transcription activator of the miR-17-92 cluster. The additional target of MYC is the transcription factor E2F1 (Figure 3), which promotes the progression of the cell cycle [34].

Expression of E2F1 promotes G1 to S phase transition in mammalian cells by activating genes required for DNA synthesis and cell cycle control. It is known that expression of the *E2F1* gene is induced by MYC. Expression of MYC is also induced by E2F1, formatting a putative positive feedback loop [35]. Furthermore, there is direct binding between E2F1 and the promoters of the miR-17-92 and miR-106a-363 clusters activating their transcription.

Observations made to date confirmed that expression of *E2F1* is negatively regulated by two miRNAs from the cluster, miR-17-5p and miR-20a, which shifts the balance from apoptotic state to proliferation, pointing to a potential anti-apoptotic role for miR-17-5p and miR-20a. In addition, miR-17-5p and miR-20a, which have identical seed sequences, inhibit translation of E2F2 and E2F3 to form an auto-regulatory loop in the E2F transcriptional network [34,36,37]. Moreover, close functional interactions between MYCC/MYCN and the miR-17-92 cluster were noted. Both MYCC and MYCN could initiate transcription by direct binding to the promoter of miR-17-92 [27,38,39].

Regulation of the miR-17-92 cluster through MYC could maintain a neoplastic state in MYC-induced tumors by sustaining autonomous proliferation and survival. However, *MYC* inactivation and the downregulation of miR-17-92, causes loss of tumor features as a result of restoration of proliferative arrest, apoptosis, and senescence [40].

Other studies showed that in cluster expression contributes an intronic A/T-rich region directly upstream of the miRNA coding region and protooncogenic kinase PIM-1 which plays an important role in the transcriptional activation of the miRNAs from the cluster. Interference of MYC, PIM-1, and E2F3 on pri-miR-17-92 levels indicated that all three proteins are important for cluster expression [41]. Additionally, recent studies confirmed the existence of interactions between transcription factors and miRNA [42]. It was suggested that the miR-106a-363 cluster is regulated by the MITF (microphthalmia-associated transcription factor) located in the cluster’s immediate vicinity [43].

## 6. First Observations of the miR-17-92 Cluster Function

For the first time an oncogenic role of miR-17-92 was suggested due to high expression of the miR-17-92a cluster host gene (*MIR17HG*) in B-cell lymphoma [32]. Comparison of tumor and control samples showed that the level of expression of the miR-17-92 cluster components increased in the case of cancer development and strongly correlates with *MYC* activity indicating for miR-17-92 oncogenic activity [44]. Until now, abnormal expression of the miR-17-92 cluster components was observed in a variety of cancers. Significant overexpression of the miR-17-92 complex was demonstrated during the development of lung cancers, especially in their most aggressive form, small cell lung cancer [45]. Inhibition of miR-17-5p and miR-20a with antisense oligonucleotides stimulates apoptosis in lung cancer cells with overexpressed miR-17-92 [46]. The increased expression of miR-17-92 members, particularly miR-17-5p was observed in colon cancer [47]. OncomiR-1 overexpression leads to much faster development of murine hepatocellular carcinoma. Moreover, overexpression of the cluster in human hepatocellular carcinoma cell cultures increases tumor cell proliferation, colonization, and invasiveness [48]. Expression of miR-17-5p was high in invasive breast cancer cells while decrease of the miRNA level inhibited cell migration and invasion [49]. 

Up to now the role of the miR-17-92 cluster is primarily focused on pathophysiological states but there are also reports of its physiological role. Studies on mouse models proved that the OncomiR-1 plays an essential role in skeletal development in mammals. Microdeletions of region containing miR-17-92 locus affected the development of skeletal defects and Feingold syndrome [39]. Deletion of miR-17-92 in mouse pancreatic β-cells showed that miR-17-92 is an important factor influencing molecular mechanisms regulating insulin secretion [50]. miR-17-92 is also required for cardiomyocyte proliferation and its overexpression protects the heart muscle from damage during a myocardial infarction [51].

## 7. miR-17-92, miR-106b-25, and miR-106a-363 in Brain Tumors

### 7.1. Glioblastoma (GBM)

Glioblastoma (GBM), the most common primary brain tumor in adults and definitely less frequent in pediatric population, is still connected with unfavorable prognosis [52,53]. The average survival time after diagnosis is approximately 12–15 months with current therapy including maximum surgical resection, radiation, and chemotherapy [54]. According to the 2016 WHO classification glioblastomas are divided into two molecularly defined subgroups based on the *IDH1/2* gene status [53,55,56]. Despite the huge progress of molecular knowledge, the GBM cells of origin are still unknown. Moreover, histological and molecular heterogeneity of this tumor indicates for diverse progenitors what makes the targeted therapy difficult [57].

Glioblastoma is the best characterized tumor of the central nervous system for the potential tumorigenic role of miR-17-92 cluster members. Molecular analyses were performed both on clinical material and human cell lines. 

In silico analyses showed that miR-17-92, miR-106b-25, and miR-106a-363 cluster members could be elevated in human gliomas including GBM. In addition, studies conducted on cell cultures derived from patient’s samples showed that miR-20a, miR-20b, miR-93 and miR-106a are up-regulated in comparison to the normal brain tissue (Table 1). 

Such observations suggest an increased activity of the E2F and MYC in cell cycle. As the E2F pathway leads to cell cycle progression during CDK4 and CDK6 activation, the CDK4/6 inhibitors were used in cell cultures. Among factors underlying the response to CDK4/6 inhibition, E2F as well as miR-20a, miR-20b, miR-93, and miR-106a were noted [58].

Expression analysis of miR-17-92 cluster members revealed that miR-17-5p, miR-92a-1, and miR-106b expressions were significantly higher in glioblastoma samples in comparison to normal brain tissue. The decrease expressions of those cellular regulators were observed during miR-17-92 inhibitors transfection of GBM cultures. The use of anti-miRs directed against all six miRNAs of the miR-17-92 cluster decreased cell viability and proliferation [59]. 

In following studies, the effect of miR-17-5p activity on cell proliferation and apoptosis of GBM cells were thoroughly evaluated. The miR-17-5p expression levels examined in 108 glioblastoma and 20 normal brain tissue samples were significantly higher in tumor specimens. In addition, there was a relationship between the level of miR-17-5p expression and the clinical outcome; the overall survival of patients with GBM with high miR-17-5p levels was significantly shorter [60].

The aim of the Song et al. study was to investigate the biological role of miR-18a in human glioma of different grade [64]. Expressions of miR-18a were significantly higher in glioblastoma tissue and glioblastoma cell lines in comparison to control brain tissue; remarkably, the expression levels increased with the rising pathological grades of gliomas. Furthermore, miR-18a negatively regulated the mRNA and protein expression of neogenin, which plays various physiological roles in normal tissues. Inhibition of miR-18a expression significantly up-regulated neogenin, reduced cell proliferation, migration, invasion, and induced cell cycle arrest [64]. 

Other studies showed that miR-19a and miR-19b were overexpressed in glioblastoma samples and cell lines in comparison with normal brain tissue, and their expression level positively correlated with tumor grade within analyzed glioma samples. Additionally, database search and luciferase reporter assay demonstrated that one of the target genes of miR-19a/b is *PTEN* which is involved in regulation of the cell cycle [66].

Similarly, upregulated levels of miR-92a were shown in glioblastoma. It was demonstrated that this miRNA contributed to the regulation of cell apoptosis and proliferations. miR-92a was upregulated in human glioma samples and cell lines compared to normal adjacent tissue. Subsequent studies on cell lines with miR-92a inhibitors showed a reduction in miR-92a level expression between 74–81% depending on the cell lines. Knockdown of miR-92a significantly increased cell apoptosis due to upregulated expression of pro-apoptotic BAX and cleaved-caspase-3 in xenograft tumors. Such observation underlined an oncogenic role for miR-92a and suggested the potential role of antimiRNA inhibitors in glioblastoma therapy. Bioinformatic analysis identified *BIM* (*BCL2L11*) which may be a target gene for miR-92a. This observation was confirmed by plasmid transfection and in vivo studies which demonstrated that the high expression of miR-92a in human glioma specimens was significantly correlated with low levels of BCL2L11 protein and a higher tumor’s grade [68]. 

Another study demonstrated over-expression of miR-25 (element of miR-106b-25 cluster) in glioblastoma samples. Cell line analyses showed miR-25 effects on proliferation, migration, and invasion. The effect was suppressed by the use of miR-25 inhibitors. Moreover, it was identified as a novel molecule target of miR-25, neurofilament light polypeptide (NEFL). miR-25 expression is inversely correlated with NEFL. Moreover, miR-25 represses the activation of the mTOR pathway by targeting NEFL which inhibited GBM cell proliferation and invasion [69].

Functional analyses of glioblastoma cell cultures revealed essential role of miR-106a (miR-106a-363 cluster) in tumor cell invasion. This observation was connected with activation of WNT signaling pathway by miR-106a which modify β-catenin cellular localization. Furthermore, miR-106a by facilitation of binding the β-catenin to the promoter regions, enhanced transcription of *MMP2*, *NANOG*, and *MYC*, as well as genes related to cell invasion *RUNX2*, *CD44*, *SOX9* and *OCT4* [71].

miR-363 was also involved in glioma progression and its expression was correlated with WHO grade. Inhibition of miR-363 expression lead to the downregulation of the MMP-2 and MMP-9 cellular factors involved in the breakdown of the extracellular matrix. Caspase 3, the main effector of apoptosis, was elevation by miR-363 inhibition while expression of BCL-2, one of the main anti-apoptotic effectors were relevant reduced. This data suggested that this miRNA may play a role in the control of proliferation, cell cycle progression, and glioma invasiveness. The potential target for miR-363 is the GAP-43 (growth-associated protein 43) which was largely expressed in developing and differentiated neurons [72].

### 7.2. Medulloblastoma (MB)

Medulloblastoma (MB) is the most frequent type of high grade tumors occurring in the pediatric population (15% to 25% of pediatric brain tumors) located in the cerebellum. Despite significant advances in therapy, it is still associated with a high mortality rate. Until now, a number of molecular prediction factors for medulloblastoma have been proposed. The current update of the WHO classification of tumors of the central nervous system distinguishes within medulloblastomas four molecular subgroups that have a prognostic and therapeutic value: WNT-activated, SHH-activated and enigmatic group 3 and group 4 also described as the non-WNT/non-SHH.

The origin of this neoplasm is still under discussion. The SHH and WNT cases arise respectively from granule neuron and dorsal brainstem progenitors. The cerebellar stem cells were suspected as the origin of group 3 tumors but origins of group 4 tumors are still unknown [55,73,74]. 

Comparative studies between human primary MBs and normal human cerebellum revealed consistent overexpression of miR-17-92 and its related paralogs (miR-106a-363 and miR-106b-25) [75]. Eleven out of 20 significantly increased miRNAs belong to this miRNA group, providing strong evidence to support significance of miR-17-92 overexpression in MBs. MiR-17-92 expression was the highest in the SHH-activated subgroup, followed by group 3 and WNT-activated cases. Analysis of miR-19a, miR-92, and miR-20a in pediatric MBs showed increased expression levels in SHH-activated MBs in comparison to those showing a lack of SHH pathway activation [67]. These results indicated for miR-17-92 cluster significance during MB formation driven by an aberrant SHH pathway and suggested a functional relationship between those molecular factors. Moreover, study performed using the mouse model of MBs underlined overexpression of the miR-17-92 [75]. Additionally, the correlation between *MYCN/MYC* and miR-17-92 expression suggested that miR-17-92 regulation may be *MYCN/MYC* dependent.

Due to the type of MBs some studies tried to elucidate connection of *MYC* family (*MYC, MYCN, and MYCL1*) expression with the aggressiveness of the tumor. In large number of observations *MYC* overexpression was correlated with a worse prognosis [76,77,78]. However, in some cases of the WNT-activated medulloblastomas survival was good despite the high *MYC* expression [79]. In the SHH-activated subgroup *MYCN* and *MYCL1* were highly expressed compared to other molecular variants. MYCN amplification in this subgroup was a marker of enhanced SHH activity and was strongly associated with poor prognosis [80]. Group 3, similarly to WNT tumors, was characterized by higher *MYC* expression, while *MYCN* was expressed at a low level [81]. Expression of *MYC* and *MYCN* in group 4 tumors was generally low in comparison to the other subgroups but mean *MYCN* expression levels were still comparatively higher than in a normal cerebellum [82]. Research on a mouse model of medulloblastoma demonstrated that myc was not only necessary for tumor initiation but was also required to maintain the growth of MB tumors [83].

Recently, a study on the biological role of miR-17-92 was performed by inhibiting miR-17 and miR-19a function using 8-mer LNA-modified antimiRs directed against their seed sequences on a mouse model of SHH-activated medulloblastoma and primary MB cells from spontaneously arisen SHH-activated MBs originated in mice. In vitro inhibition of miR-17 and miR-19a seed families resulted in decreased proliferation of tumor cells. Mice treatment with antimiR-17 and antimiR-19 reduced tumor growth and prolonged the survival of animals [84].

### 7.3. Ependymoma (EP)

Ependymal tumors are the least common brain tumors in children, comprising 2% to 9% of all central nervous system tumors in pediatric population [85]. Ependymomas are WHO grade II or III tumors arising from radial glial cells in the ependymal lining of the ventricles or spinal cord. Lately, several gene expression profiling analyses of ependymomas shed a new light on understanding of the genetic of these tumors and open the new possibilities in search for the crucial biomarkers. It is hoped that the results of that studies could help in better differentiation, risk stratification, and adjusting therapy for children with ependymomas.

In the literature there were no studies concerning the role of the entire miR-17-92 cluster or its paralogs in ependymal tumors. The reports referred only to a single miRNA analysis. Birks et al. investigated miRNAs expression in pediatric brain tumors including eight ependymomas and revealed significantly higher expression of miR-25 in tumors compared to normal pediatric brain samples [70]. Costa et al., in their research, compared the miRNA expression profile of thirty-four ependymoma samples to normal brain controls. Among 23 overexpressed miRNAs there were components of miR-17-92 cluster and paralogs, miR-17-5p, miR-19a, miR-19b, miR-20a, and miR-106b [61]. Comparative analysis of miRNA profiles between childhood ependymomas performed on the basis of miRNA profiling with subsequent validation showed that the expressions of miR-17-5p, miR-19a, and miR-106b were strictly connected with tumor grade and patient outcome [86]. 

### 7.4. Pilocytic Astrocytoma (PA)

Pilocytic astrocytoma (PA) is the most common low grade pediatric brain tumors, comprising 21% of tumors in children under the age of 14 years and 16% at age 15–19 years. PAs are generally well characterized and are slow growing tumors [87].

Little is known about the potential role of miRNA alterations in pilocytic astrocytomas. Comparative studies between pediatric PAs and normal tissue revealed significant changes in the expression of various miRNAs. Several miRNAs were found to be upregulated or downregulated [88,65]. Among the overexpressed ones, there were miR-18a (miR-17-92 cluster), miR-18b, and miR-106a (miR-106a-363 cluster) [89].

## 8. OncomiR-1 and Its Paralogs in the Sympathetic Nervous System Tumors

### Neuroblastoma (NB)

Neuroblastoma (NB) is a solid tumor derived from the sympathetic nervous system. It occurs in the superior cervical, paraspinal, and celiac ganglia, however the majority of cases arise in the adrenal glands. NB usually affects young children which constitutes half of solid tumors. The majority of cases are diagnosed after the second year of life with the average age at diagnosis being about 18 months [90,91]. The most aggressive tumors were characterized by a variety of genetic aberrations, including *MYCN* amplification, 1p chromosome deletion, and 17q chromosome imbalance. *MYCN* amplification occurred in 25% of cases and correlated with aggressive phenotype and treatment failure [92].

In the neuroblastoma cells, the increased *MYCN* levels induced expression of the miR-17-92 cluster at transcriptional level by direct binding to its promoter. Among the miRNAs belonging to the miRNA 17-92 complex, miR-17-5p, and miR-20a were overexpressed. In vitro and in vivo studies showed that the miR-17-92 cluster enhanced cell proliferation and promoted tumor growth. The use of antagomiR-17-5p not only caused a cell cycle block but also lead to apoptosis [62].

There are studies which demonstrated that overexpression of miR-92, miR-106a, miR-17-5p, and miR-93 in neuroblastoma were functionally linked to *MYCN* amplification. These miRNAs were correlated with *MYCN* amplification as well as *MYCN* activation [63]. Samaraweera et al. indicated existence of an inverse relationship between *MYCN* expression and miR-17-5p levels. miR-17-5p was induced by *MYCN* and reciprocally binds to the gene 3′UTR region to downregulate its expression, creating a negative-feedback loop in *MYCN*-amplified neuroblastoma cell lines. Additionally, the neuronal-specific RNA-binding protein HUD was involved in this regulation, through binding and stabilizing the *MYCN* transcript. It was demonstrated that HUD and miR-17-5p compete for adjacent overlapping binding sites in the 3′UTR of *MYCN*, exerting antagonistic effects on *MYCN* mRNA stability and subsequent protein expression [93]. Another protein regulated by the miR-17-92 cluster is Dickkopf-3 (DKK3). This member of the DKK family of WNT antagonists acts as a tumor suppressor in a wide range of tumors. 

In neuroblastoma, DKK3 seemed not to affect WNT signaling but functioned beyond mere canonical WNT inhibition. DKK3 expression levels were inversely correlated with the expression of members of the miR-17-92 cluster. A strong decrease in mRNA and protein level was observed after miR-17-92 overexpression, and it was suggested that miR-19b and miR-92a were responsible for this effect. It has also been shown that these miRNAs molecules are the missing link between MYCN and DKK3 [94,95]. 

Moreover, miR-17-92 was a potent inhibitor of TGFB1 signaling in neuroblastoma. miR-17-92 expression was inversely proportional to the expression of TGFB1 responsive genes. The TGFB1 signal was reduced in aggressive neuroblastoma tumors with high-expression of miR-17-92. Activation of miR-17-92 triggered the downregulation of many key effectors along the TGFB1 signaling cascade, as well as direct inhibition of TGFB1-responsive genes. It has been shown that no single miRNA but the whole cluster mediated the repression of TGFB1-signaling in neuroblastoma cells [96]. The role of NHR (nuclear hormone receptors) which play an important role in controlling the fate of neural stem cells and neural differentiation, was investigated with respect to activity of the *MYC* pathway. It was demonstrated that miR-17-92 cluster regulated by *MYC* inhibited genes encoding NHRs. Up until now, seven members of the NHR superfamily were identified as putative targets of the miR-17-92 cluster [97].

## 9. Conclusions

A variety of miRNAs interact with many cellular networks and pathways as regulators of physiological and pathophysiological processes. So far, the role of non-coding sequences including miR-17-92, miR-106b-25, and miR-106a-363 clusters was also documented in many cancers. It is postulated that miRNA expression correlates with histological origin of the tumor and stage of the diseases. Supposing miRNA expression could be more adequate at classifying tumors than mRNA profiling, the meaning of miRNA alteration in pathology of tumors, including brain lesions, seems to be growing. However, miRNA genes undergo complex alterations that change miRNA sequences and/or activity, have more than one target, and therefore the miRNA analyses could be difficult to interpret. According to that miRNA expression, it should be explored in detail due to their usefulness during future diagnostic approaches, estimation of outcomes, and plausible treatment decisions. 

## Figures and Tables

**Table 1:** Overexpression of miRNAs from miR-17-92, miR-106a-25, and miR-106b-363 clusters in brain tumors and neuroblastoma.

Cluster Name	miRNA	GBM	MB	EP	PA	NB
miR-17-92	miR-17-5p	[59,60]		[61]		[62,63]
	miR-18a	[64]			[65]	
	miR-19a	[66]	[67]	[61]		
	miR-20a	[58]	[67]	[61]		[62]
	miR-19b-1	[66]		[61]		
	miR-92a-1	[59,68]	[67]			[63]
miR-106b-25	miR-106b	[59]		[61]		
	miR-93	[58]				[63]
	miR-25	[69]		[70]		
miR-106a-363	miR-106a	[58,71]			[65]	[63]
	miR-18b				[65]	
	miR-20b	[58]				
	miR-19b-2	[64]		[61]		
	miR-92a-2	[68]	[67]			[63]
	miR-363	[72]				

GBM—glioblastoma; MB—medulloblastoma; EPN—ependymoma; PA—pilocytic astrocytoma; NB—neuroblastoma. In square brackets there are numbers of references.
